# Recursive Combination Has Adaptability in Diversifiability of Production and Material Culture

**DOI:** 10.3389/fpsyg.2018.01512

**Published:** 2018-09-19

**Authors:** Genta Toya, Takashi Hashimoto

**Affiliations:** School of Knowledge Science, Japan Advanced Institute of Science and Technology, Nomi, Japan

**Keywords:** recursive combination, hierarchical structure, evolutionary simulation, action grammar, evolutionary linguistics, tool manufacturing

## Abstract

It has been suggested that hierarchically structured symbols, a remarkable feature of human language, are produced via the operation of recursive combination. Recursive combination is frequently observed in human behavior, not only in language but also in action sequences, mind-reading, technology, etc. in contrast, it is rarely observed in animals. Why is it that only humans use this operation? What is the adaptability of recursive combination? We aim (1) to identify the environmental feature(s) in which recursive combination is effective for survival and reproduction, and that has facilitated the evolution of this ability, and (2) to demonstrate the possible evolutionary processes of recursive combination. To achieve this, we constructed an evolutionary simulation of agents that generated products using recursive combination and used the results to explore the types of fitness functions (that reflect the kinds of adaptive environments) that give rise to this ability. We identified two types of adaptability of the recursive combination: (1) diversifiability of production and (2) diversifiability of products. Through the former, recursive combination promotes robustness against failure of production caused by inaccurate manipulations or irreversible changes. In an environment in which diversified products are preferable, sharing a portion of the production process for these products entails producing multiple products in which recursive combination plays a key role. We suppose that recursive combination works as a driving force of material culture. Finally, we discuss the possible evolutionary scenarios of recursive combination that is later generalized to encompass many aspects of human cognition, including human language.

## Introduction

**Figure 1:**
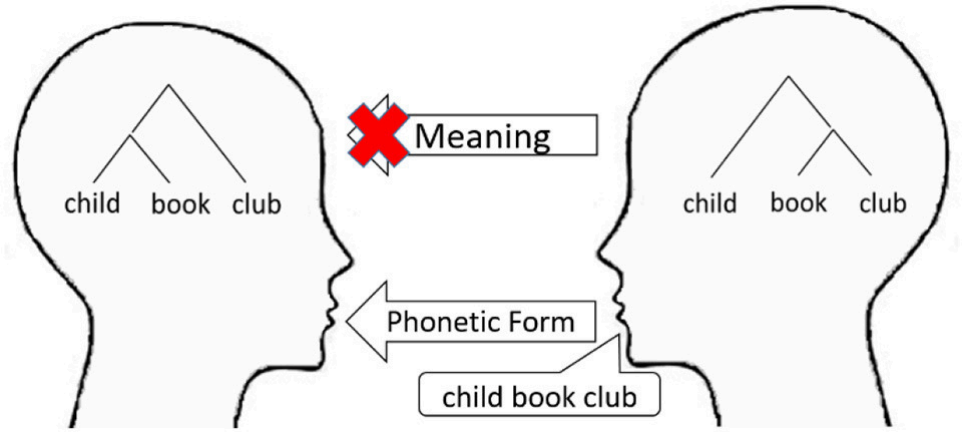
Structure dependency, which may cause misunderstanding in communication.

One of the most remarkable features of human language is its hierarchically embedded structure (Chomsky, 1957). Although both animal calls and human languages use one-dimensional sound signals in communication, words are organized hierarchically into sentences in the latter unlike in the former (Hauser et al., 2002). This feature recognizes the fact that the meaning of a sentence depends on its hierarchical structure and not on word order alone (Figure 1). This structural dependency may cause misunderstandings in communication, since the structure determining the meaning is not expressed unambiguously in a linear word sequence but only via interpretation (involving selections from multiple possibilities inside the speaker's and the listener's minds). If the adaptability of language contributes to information transmission and mutual understanding, for example, to promote cooperation in a group, structural dependency will cause a disadvantage. We need to consider the adaptive value of language equipped with hierarchically embedded structures and structural dependencies in the period of the language's origin.

An important perspective was proposed by Kirby (2017) that cultural effects have a stronger impact than biological effects on the origin of linguistic structure. Kirby claims that human behaviors developed rich systematic structure such as recursion, compositionality and hierarchical structure to be expressive. Although we agree with this claim, we need to clarify the origin of linguistic ability, that is, operation, to construct rich structures.

**Figure 2:**
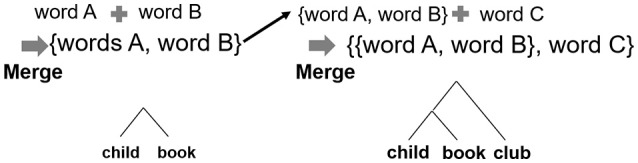
Syntactic operation for making sentences referred to as *Merge*.

Studies on hierarchical structure as the fundamental aspect of language (from the perspective of generative grammar) assume that recursive combination capacity, defined as the capacity to combine two items into a set, is the most important ability required for constructing hierarchical structures. This capacity is applied to enable a recursive syntactic operation. The different hierarchical structures are created by two types of combination, recursive combination and non-recursive combination. On one hand, theoretical linguists suggest that *Merge* (Figure 2) is a set-formation operation that can be used to create an unbounded number of sentences through its recursive application (recursive Merge) (Chomsky, 1993, 2013; Everaert et al., 2015). On the other hand, *Unification* is also a set-formation operation that has been proposed by other researchers (Jackendoff, 2002, 2011). Jackendoff claims that *Recursion* is found everywhere in higher cognition; therefore, operations such as unification that can be applied to language expression and to other mental structures are needed. The important point is that both Merge and Unification share recursive combination as the core of the operations.

Recursive combination of language has been hypothesized as a human-unique trait (Hauser et al., 2002; Fitch and Hauser, 2004; Fujita, 2009, 2016). How did recursive combination and structure-dependency originate? Structure-dependency greatly increases the capacity for ambiguity in language communication. Thus, it is unreasonable to assume that recursive combination evolved to meet the needs of simple communication with one-to-one mapping between meanings and forms.

Hierarchical structure and recursive combination are described in other domains as follows:

- In action, the complex action sequences are represented as a hierarchical structure including the final goal and subordinate goals (Jackendoff, 2011). This structure is made up of three parts: the HEAD, the PREPARATION, and the CODA. For instance, when we use an automatic drip coffee maker, the action sequence “put coffee in machine” is made up of smaller steps, such as preparing the filter (preparation), putting the coffee in (head), and closing the filter (coda). “Putting the coffee in” also includes steps such as getting the coffee can out of the freezer (preparation), measuring the coffee (head), and putting the coffee away (coda), and each of these steps can be broken down further.
- In technology, new technology is constantly derived from components that already exist; in turn, these new technologies offer themselves as possible components for the construction of further new technologies (Arthur and Polak, 2006; Arthur, 2009). Technology, which is the collection of mechanical devices and methods available to a culture, becomes assimilated and combined. Therefore, this self-production is the combination of combined objects.
- In music, “discrete structural elements such as beats are hierarchically combined into larger groups according to rules” (Asano and Boeckx, 2015, p. 2). Musical metrical structures have a hierarchical structure that include the recursive embedding of beats into beats. The existence of hierarchical structure in meter has received support from neurological evidence (Bouwer et al., 2014).
- In the Theory of Mind, intentionality can be correlated to hierarchical structure with recursive embedding. Intentional states provide a natural platform for communication through mentalizing capacity. In this way, intentionality forms a naturally reflexive hierarchy (i.e., I suppose that you intend that I believe that you want me to understand that …). Representing another person's mental state is thus inherently recursive (Oesch and Dunbar, 2017).

These frameworks indicate that human behaviors and mental or physical structures can be treated as combinatorial objects. Boeckx (2017) claims that the neural basis of recursion is realized from the pairing of the fronto-parietal and fronto-temporal networks. He takes it that although both networks may be of the finite-state variety, pairing two finite-state devices could have the effect of boosting computational possibilities. Instead of operating on one-dimensional sequences, one now operates on two-dimensional tree representations. The fronto-parietal network may have the role of the global workspace as proposed by Dehaene et al. (1998). The global workspace is inherently hierarchical: It sits on top of modular networks of other cognitive domains and acts as a chunking device in a sequence producer. If this device is to be integrated with another sequencing machine, sequences of sequences would naturally emerge. Once this network is established, a variety of cognitive domains co-opt and account for other aspects of human-specific cognition (Boeckx, 2017). In this paper, the domain-general characteristics of discrete object combination and recursion are focused upon these.

**Figure 3:**
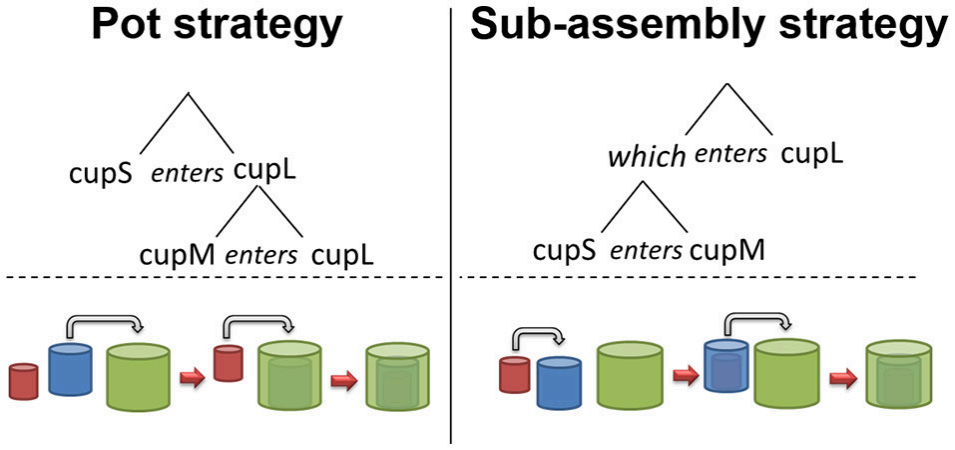
Two strategies of action grammar (adapted from Greenfield et al., 1972).

When do object recognition and its recursive manipulation advance? Recursive combination has been observed in the object manipulation of animals and has also been researched in a cup-combining experiment with human infant participants (Greenfield et al., 1972). Greenfield posited the notion of a *grammar of action*, or in other words, a set of syntactic rules for behaviors such as object manipulation. Sequential behaviors are classified into two strategies in the framework of action grammar: the *pot strategy* and the *sub-assembly strategy*, visualized below (Figure 3) with the manipulation of cups used to illustrate object manipulation.

- Pot strategy: Repeated combination. Multiple active objects act on a single static object.
- Sub-assembly strategy: Recursive combination. Two objects are combined into a pair, which is then manipulated as a single unit in the combination.

It has been noted that sub-assembly strategy or other equally similar behavior such as tool-making is rarely observed in animal behavior (Greenfield, 1991; Conway and Christiansen, 2001). Therefore, it is assumed to be a precursor of recursive combination in syntax (Maynard Smith and Szathmáry, 1995; Fujita, 2009, 2016). Although Chomsky and Berwick (2015) insist that the recursive combination, Merge, abruptly appeared at some time in human evolution, we assume that it was a gradual evolutionary scenario. We further presume that object manipulation of a physical entity was a pre-adaptation to recursive syntactic operation, and the target of manipulation was qualitatively generalized. This is a reasonable hypothesis derived from the following evidence in addition to the results of comparative cognitive experiments and analysis in archeology:

- Children develop the ability to perform sub-assembly strategy and hierarchical language structure almost simultaneously (Greenfield et al., 1972). Further, the order of development in object manipulation is similar to syntactic operation. When developing object manipulation, children acquire pot strategy first and then acquire sub-assembly strategy at a later stage. During the development of syntactic operation, they learn to combine two words to form a higher order grammatical relation as an early operation. Later, children discover the grammatical complexity of adjectives and nouns that combine to form a superordinate noun phrase that enters a still higher order combination with a verb (Brown, 1973; Greenfield, 1991).
- Archeological evidence of the use of recursive combination to make stone tools from 0.28 million years ago (mya) encourages this hypothesis (Moore, 2010, 2011). Moore (2011) compares the production methods of stone tools of the Oldowan and Acheulian types and points out that the difference lies in the hierarchical structure of the action sequences. The Oldowan tool is generally produced by making stone flakes from a stone core. Making stone flakes from a stone core is called the flaking process. The Acheulian tool is produced by shaping a large stone flake in combination with this flaking process. This production method reflects hierarchically organized higher order intention and suggests that recursive combination of action sequences is followed. In addition, Stout (2011) illustrates stone tool-making using tree diagram. Stout shows that hominins used recursive combination in a production sequence with sub-goals when making stone tools. These are dated earlier than the appearance of symbolic behavior in human evolution (Mithen, 1996). It suggests that the recursive combination of objects pre-dated the recursive combination of lexical items.

These findings suggest that humans might have acquired recursive combination (that is a different evolutionary effect of language on communication such as sharing information) through an action sequencing process such as tool-making. Henceforth, we term the pot and sub-assembly strategies *non-recursive combination* and *recursive combination*, respectively.

The hypothesis that social recognition and population size cause recursive mental structure is reasonable because it assumes an evolutionary continuity carried over from non-human animals (Dunbar, 2009; Oesch and Dunbar, 2017). According to this hypothesis, recursive thinking became the necessary cognitive scaffolding. Dunbar claims that recursion in the language structure is boot-strapped by a primitive mentalizing ability as evidenced by an experiment that investigated correlation between recursive syntax and intentionality. However, it must be noted that *recursion*, which is often assumed to be the subordinate clause in a sentence is not equal to “recursive combination” in this paper. Recursive combination means “combination of combined objects,” thus this interpretation of recursion can also be applied to mental object manipulation like mind-reading. We will elaborate on this point later in the Discussion section.

It is most important that we answer the following questions. What is the evolutionary process of recursive combination? What does the adaptability of the recursive combination consist of, if the process is adaptive evolution?

According to Tinbergen (1963), adaptability (which is effectiveness in survival and reproduction) is an important aspect used to explain the characteristics of animals. Although the adaptability of the human ability of recursive combination has been investigated in comparative cognitive science; similar traits have not yet been discovered. Furthermore, the phenomenon of evolution can only be observed in living things that have rapid generation alternation. The evolution of higher cognitive ability is not that easily studied. This problem can be solved partially by using simulations (Hashimoto, 2001). The advantage of simulation is that it allows the elaboration of hypotheses and the consideration of evolutionary processes. This is enabled by repeating the experiments in a constructive environment on a phenomenon that is difficult to observe empirically. It is not possible to prove a hypothesis solely by using this method. However, we can explain the process of the generation of a system (in this research, capacity of agent and ecological environment) causing a specific phenomenon (the evolution of recursive combination) by reproducing the phenomenon by implementing and operating a model derived from the hypothesis.

In this paper, we study the evolutionary process and adaptability of recursive combination using evolutionary simulations. The objectives are (1) to demonstrate the conditions in which recursive combination could have evolved, and (2) the possible evolutionary processes by which recursive combination could have evolved. We will claim that recursive combination has two adaptabilities; the diversifiability of production methods that promotes the secure manufacturing of the target product and the diversifiability of products by the reuse of parts of manufacturing processes that are already acquired. Two factors promote these adaptabilities: (1) extending the time available for making products, and (2) decreasing the cost of object manipulation. As a possible evolutionary process, it is necessary to increase the opportunity for production and reduce the manipulation cost before the evolution of recursive combination.

The rest of this paper is organized as follows: (1) The simulation model to examine whether agents evolve to be capable of recursive combination is described in section Materials and Methods. (2) The simulation results and resulting considerations for the model are presented in section Results. (3) A discussion based on the simulation results in consideration with other results is delivered in section Discussion. (4) The conclusion is delivered in section Conclusion.

## Materials and methods

In this section, (1) the concepts and mechanisms of genetic algorithm (GA) and evolutionary simulation are introduced; (2) the model of object manipulation used in this paper is explained; (3) we describe how recursive and non-recursive combinations are modeled; (4) to illustrate evolutionary simulation of object manipulation, we describe the encoding of a state transition table onto a gene and also the simulation flow; and finally, (5) three fitness functions in the evolutionary simulation are posited.

### Evolutionary simulation for investigating adaptability

Evolution has three basic factors; (1) *Variation* meaning that there are groups with different traits. (2) *Selection* meaning that variation causes differences of survival probability depending on the environment. (3) *Inheritance* meaning that the traits aiding in the survival of individuals will be passed on to the next generation. These mechanisms can be written as a sequential procedure that is the genetic algorithm.

The genetic algorithm is constructed from the following processes:

- Generation of population (variation): Generate individuals having different genes representing different traits.
- Evaluation depending on fitness function (selection): Evaluate the genes and give them fitness values according to fitness function. The fitness function is formed and abstracted from the ecological environment.
- Reproduction with crossover and mutation (Inheritance with modification): Pass on the genes of individuals with a high fitness value to the next generation. Genes in the next generation are modified by the process of gene crossover in the parents, and mutation.

If the fitness function is presented as a problem, then the genes are the optimized solution to this problem by a cumulative process.

Typically, a genetic algorithm is used to search for (quasi-)optimal solutions according to a fitness function representing an optimization problem. However, we intend to identify fitness functions having recursive combination (as an abstract operation) as their solution. Therefore, we define the candidates for the fitness functions by considering the ecological meanings of recursive combination, i.e., the evolutionary processes and adaptability are examined by evolutionary simulations. It is not our intention to model biological evolution directly, and this simulation does not reproduce the process of human evolution.

### Model of the object combination operation

#### Abstraction of recursive combination and non-recursive combination

Prior to designing the model, we considered the computational difference between recursive combination and non-recursive combination. The crafting of a stone spear from diverse materials such as wood for the shaft, a chiseled stone for the head and adhesives used to bind everything together is a good example. Such tools had been made in 0.2 mya (Wymer, 1984). When non-recursive combination is performed, one object is combined repeatedly, i.e., the builder attaches the base of the stone edge to the wooden shaft, and fixes it using an adhesive. Thus, this operation needs both a finite set of states that is expressed as an object and a transition function that is expressed as a combination. When recursive combination is performed, combined objects combine to form another object, i.e., the builder attaches the base of the stone edge to the part where the adhesive was applied beforehand on the wooden shaft. Therefore, this operation needs two finite sets of states (the state for combining and the state for storing) and the transition functions that are expressed as storing and retrieving.

#### Agent performing object manipulation

An agent performing object manipulation to manufacture products is modeled using an automaton with a stack. The aim of the agent is to make products by combining the objects (hereinafter, an elemental object is represented by a letter such as *A* or *B* and a combined object by concatenating letters, such as *AB* or *ABC*). An agent is equipped with a workspace in which objects are combined and a stack in which objects are stored temporarily from the workspace. The objects correspond to the cups in the experiments of Greenfield (1991) and Matsuzawa (1986); two or more objects cannot exist in the workspace simultaneously, and this is true for the stack as well. There are any number of objects of the same type in a set of elemental objects; thus, it is possible to make a product including multiple instances of the same type of object, such as *AAB* or *AAA*. Once combined, the objects are treated as one object and cannot be separated into two objects.

In this simulation, in order to clarify the difference between recursive combination and non-recursive combination, both combinatorial operations can produce the same set of objects by assuming that a combined object has a linear structure with directionality. Therefore, an object is added at the end of another (elemental or combined) object.

The agent performs the following four actions, depending on the state of its workspace and stack:

- Get: Combine an elemental object at the end of an object in the workspace (when there is an object in the workspace) or place an elemental object in the workspace (when the workspace is empty). The elemental object is given randomly from the set of elemental objects.
- Stop: Designate an object in the workspace as a finished product. This can be executed only when the stack is empty.
- Push: Store an object in the stack; the workspace becomes empty. For simplicity, this can be executed only when the stack is empty.
- Pop: Retrieve an object from the stack. The retrieved object is combined at the end of an object in the workspace (when there is an object in the workspace) or the retrieved object is placed in the workspace (when the workspace is empty). The stack becomes empty after this action.

If multiple actions are possible in a state, one action is randomly chosen.

The initial state for the agent features an empty workspace and stack. Product-making is the process of state transitions of combined objects from the initial state to the final state. If there is an object in the stack, the agent is accepted as being in the process of production; the stack must be empty at the final state. There are *k* types of elemental objects, and an agent can make products composed of any number of elemental objects up to the maximum length, *l*, hereinafter, the maximum length of the product. The two combining actions, Get and Pop, are limited to avoid producing a combined object longer than *l*. If an agent cannot perform the Stop action when the length of the combined objects in the workspace becomes *l*, this production process is a failure, and a new production process begins from the initial state. An agent can make any number of products within the upper limit of the number of manipulation steps, which sets the agent's lifetime.

In this model, two strategies, non-recursive combination and recursive combination, are formalized, respectively, as follows:

- *Non-recursive Combination*: An agent combines an elemental object with another elemental object in the workspace. The stack is not used or stores an uncombined object only.
- *Recursive Combination*: An agent combines an elemental or combined object with a combined object that has been combined in advance and stored in the stack. Stack operations to store and retrieve the combined object are necessary.

Note that the following operations are not recursive combinations:

- Pop an elemental object stored in the stack to combine with an elemental or combined object in the workspace.
- Pop a combined object stored in the stack to place it in the empty workspace.

#### State transition table

A state transition, effected by performing an action, is expressed as:

(1) $$\begin{array}{l} \left(\text{stack },\text{ workspace}\right)\overset{\text{action}}{\to }\;\left(\text{stac}{\text{k}}^{{\;}^{\prime }},\text{ workspac}{\text{e}}^{{\;}^{\prime }}\right). \end{array}$$

**Figure 4:**
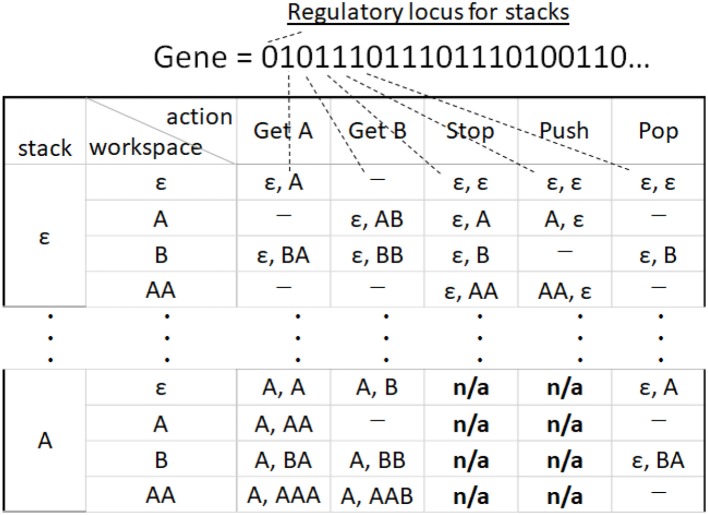
Example of part of a state transition table. The number of the types of elemental object, *k* = 2. A corresponding gene code is shown above the table. The first bit of gene is a regulatory locus for stacks.

The behavior of a particular agent is defined by the state transition table shown in Figure 4. The state transition table describes a transition of a finite number of states, in our paper, workspace and stack, of the agents. In Figure 4, the two columns on the left are the state of the stack and of the workspace, that is, the left-hand side of (1). The five columns on the right are the actions. The destination of the transition after each action, corresponding to the right-hand side of (1), is indicated in each box as the states of the stack and the workspace. The symbol “ε” signifies nothing in the stack or in the workspace; that is, it represents an empty state. An instance of “–” indicates that the agent cannot perform this transition, while “n/a” indicates that the transition is forbidden due to a non-empty stack. If more than one destination is provided, one is selected randomly. Both the number of workspace states and stack states are

$$\begin{array}{l} 1+S\;, \end{array}$$

where

$$\begin{array}{l} S=\overset{l}{\underset{l^{\prime }=1}{\sum }}k^{l^{\prime }} \end{array}$$

is the size of the combinatorial space, and the number of actions is (*k* + 3). The number of *n*/a's is

$$\begin{array}{l} 2S\left(1+S\right)\;. \end{array}$$

Therefore, the total size of the state transition table is

$$\begin{array}{l} \left(k+3\right){\left(1+S\right)}^{2}-2S\left(1+S\right)=\left(1+S\right)\left\{\left(1+k\right)+k+3\right\}\;. \end{array}$$

**Figure 5:**
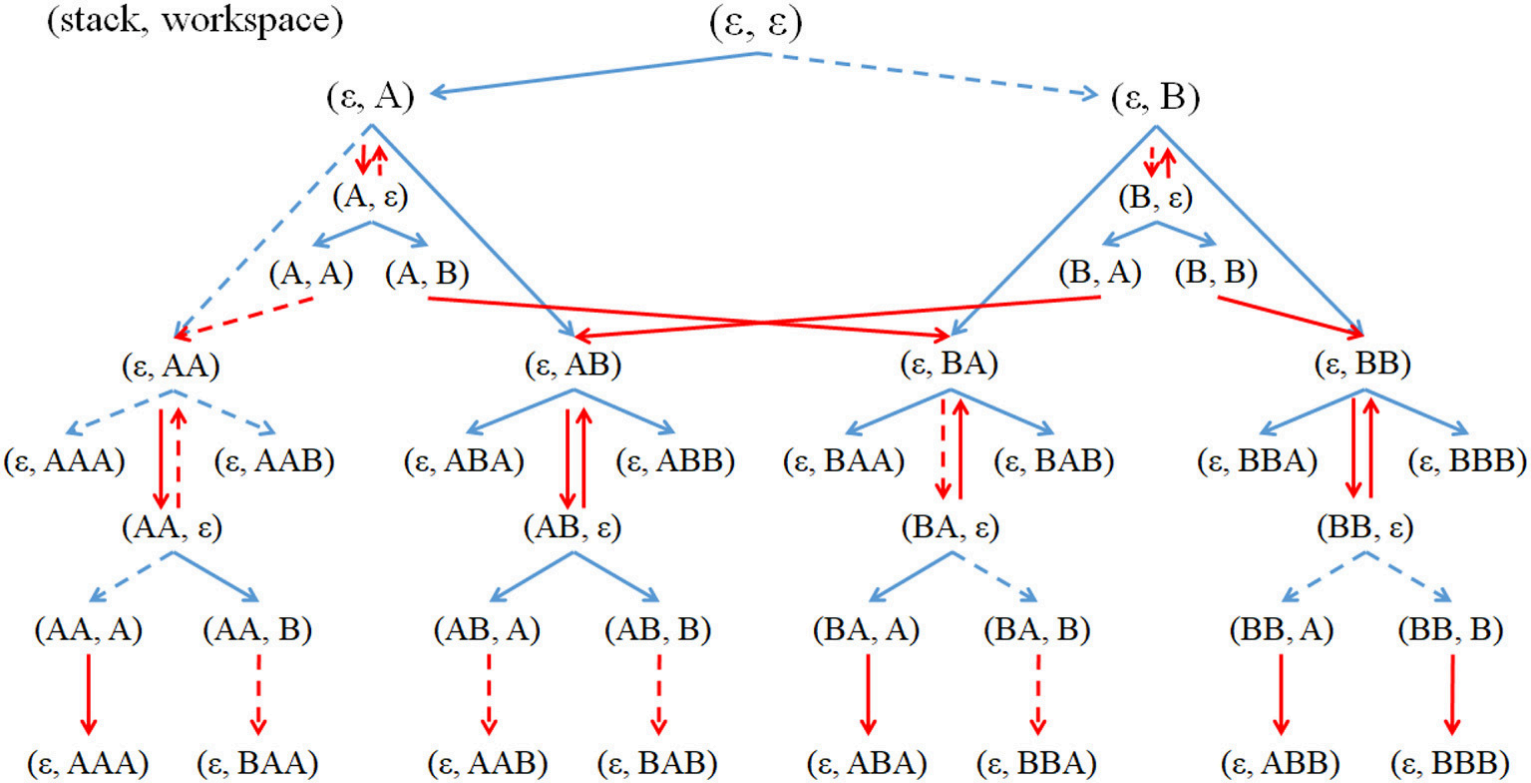
Partial state transitions using Get actions (blue arrows) and using stack actions Push and Pop (red arrows) when the number of types of elemental objects, *k* = 2, and the maximum length of each product, *l* = 3. The notation (x, y) means that x is the stack state, and y is the workspace state. An elemental object is represented by A or B and a combined object by concatenating letters, as in AB or BAA. Dashed lines indicate that the agent could not perform the transitions.

Figure 5 provides examples of state transitions corresponding to the state transition table in Figure 4. For an example of the state transitions, when an agent has states where the workspace is ε and the stack is also ε (as seen in columns 1 and 2, row 1 in Figure 4; as at the top of Figure 5), if the agent performs Get *A*, the agent will have a state where the workspace is *A* and the stack is ε (as seen in columns 1 and 2, row of workspace 2 and stack 1 in Figure 4; as seen at the left top of Figure 5). Then, if the agent performs Pop, the agent will have states where the workspace is ε and the stack is *A* (as seen in columns 1 and 2, row of workspace 1 and stack 2 in Figure 4; as seen under the top left of Figure 5). The same product can be manufactured either by using or by not using stacks, but production using stacks require more steps than the latter process.

### Model for evolutionary simulation

#### Gene encoding of transition table

The state transition table of the agent is encoded into a gene with a binary string, as shown in Figure 4. If a transition is possible, the corresponding box in the state transition table is filled; in such a case, the locus is one. If a transition is impossible, the box is “–” then the locus is zero. Boxes showing “*n*/a” are not encoded into a gene. There is a regulatory locus for stacks. If it is zero, agents cannot use any stacks even if loci for Push and Pop are on^1^. As can be seen from the figure, for an agent to be equipped with a stack that can store all possible objects, all loci corresponding to Push and Pop and the regulatory loci must be turned on in the agent's gene.

#### Simulation flow and selection mechanism

In an evolutionary simulation, the initial population's gene is generated as all loci are zero for all agents. Each agent performed production according to the state transition table encoded in its gene; the fitness of each agent is evaluated depending on the results of its production. The fitness function is defined in the following subsection.

For generation turnover, two parents are selected from the top 10% with a rank selection according to fitness values, and two offspring are produced using a one-point crossover. This process of selection and reproduction is repeated until the number of offspring reached a predefined population. Thereafter, bit inversions occur as mutations with a locus in each agent's gene in the next generation.

Although this is not a biologically plausible implementation, this design is adopted because the aim is to identify the role of recursive combinations.

#### Fitness function

The evolutionary process and evolvability of recursive combinations under each fitness function were examined by evolutionary simulations. The following three fitness functions were set.

- Making any product:
  (2) $$\begin{array}{l} F_{\text{I}}\left(t\right)=\underset{\text{all }x}{\sum }n_{x}^{i}\left(t\right), \end{array}$$
  where *x* represents a product composed of up to *l* elements and $n_{x}^{i}\left(t\right)$ is the number of times the product *x* is produced by agent *i* at generation *t*. The fitness function *F*_I_ is based on the expectation that recursive combination is used in making many products.
- Making a specific product:
  (3) $$\begin{array}{l} F_{\text{II}}\left(t\right)=n_{x}^{i}\left(t\right), \end{array}$$
  where *x* represents a product which is the longest, that is, *l*, and consists of the most number of types of elemental objects, namely, *k*. This fitness function is based on the fact that human made products have become increasingly complex in structure (Stout et al., 2008; Arthur, 2009). We choose a target product such as *ABAB* (*k* = 2, *l* = 4) or *ABCAB*C (*k* = 3, *l* = 6).
- Making products as diverse as possible:
  $$\begin{array}{l} F_{\text{III}}\left(t\right)=\;\underset{\text{all }x}{\sum }\delta \left(n_{x}^{i}\left(t\right)\right),\; \end{array}$$
  (4) $$\begin{array}{l} \delta \left(n_{x}^{i}\left(t\right)\right)=\{\begin{array}{c} 1,\;n_{x}^{i}\left(t\right)\geq 1\\ 0,\;n_{x}^{i}\left(t\right)=0 \end{array}. \end{array}$$

This fitness function is based on the fact that humans make increasingly diverse products (Arthur, 2009). We expect that manufacturing many types of products encourages an agent's survival and reproduction, while manufacturing the same product does not.

Although the manipulation steps for making one product are not explicitly expressed in these fitness functions, they nevertheless indirectly influence agent fitness because an upper limit of the number of manipulation steps is set. Thus, when an agent requires a considerable number of manipulations to make one product, the number of products made decreases and the agent's fitness is reduced.

## Results

The purpose of this evolutionary simulation is to clarify the adaptability of recursive combination to demonstrate the conditions of the ecological environment and the process of evolution. In the first subsection, we show the simulation results in the three fitness functions introduced above, at first by setting the number of types of elemental object *k* = 2 and the maximum length of product *l* = 6. Then, the dependencies of these results on the parameters, *k* and *l*, are illustrated. These analyses suggest that recursive combination has two kinds of adaptabilities. In the second subsection, considerations based on the adaptabilities are used to modify the fitness functions to add cost factors that may affect the evolution of recursive combination. It is expected that the cost of manipulation influences negatively the evolution of recursive combination because it requires a greater number of manipulation steps than non-recursive combination. We also investigated the influence of a possible failure of operation on the evolution of recursive combination. We considered the evolutionary mechanism of recursive combination only on the simulation in this section of the paper. The cognitive or linguistic interpretations about the simulation results are considered in the Discussion section.

The parameters are summarized as shown in Table 1. The population size is 100, and the upper limit of manipulation steps is set at 10,000, which does not influence the results unless it is too small. Simulation results were taking 200 runs in each parameter. In this section, hereinafter, recursive combination, non-recursive combination, and the agent using recursive combination are called *RC, non-RC*, and *RC agent*, respectively.

**Table 1:** List of simulation parameters.

**Name of parameter**	**Symbol**	**Value**
Population size	–	100
Upper limit of manipulation	–	10,000
Number of types of elemental object	*k*	1~4
Maximum length of product	*l*	3~8

### The fitness function for which the recursive combination is adaptive

#### Making any products

**Figure 6:**
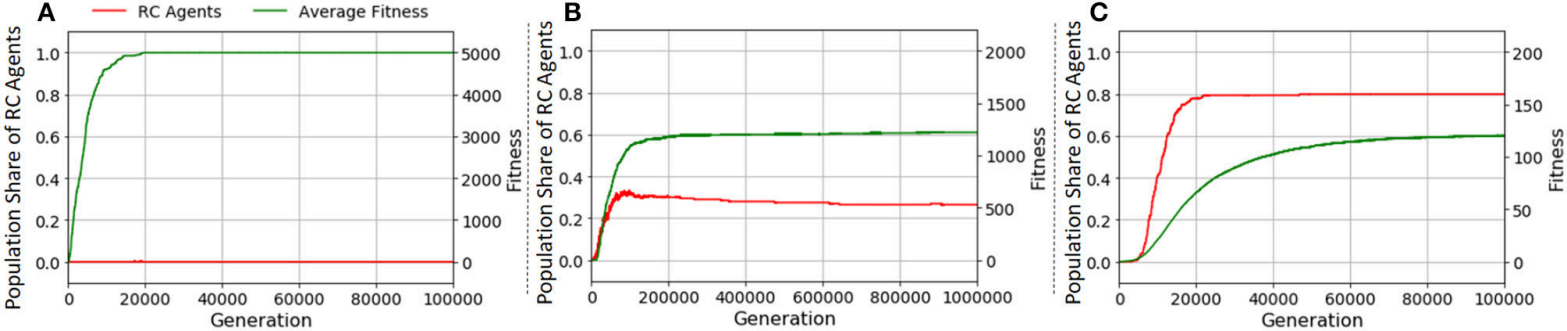
Transitions of the population share of RC agents in **(A)** *F*_I_, **(B)** *F*_II_, and **(C)** *F*_III_ (average of 200 runs). The x axes denote generation. The y axes on the left denote the population share of RC agents (red line), and those on the right denote average fitness over the population (green line).

With the fitness function *F*_I_, RC agents did not evolve in all the 200 runs as shown in Figure 6A. Since the fitness function *F*_I_ encourages the act of making any product, agents gained fitness by repeatedly making specific simple products over many production trials. The average fitness is 5,000 with the upper limit of manipulation steps set at 10,000. This fitness value indicates that agents make products containing only one element such as *A* or *B*, using Get and Stop actions, that is, two manipulations, and RC is not used as shown. The number of types of product is one with slight fluctuations. This means that the population is mostly occupied by agents making products with one elemental object. This result suggests one reason that RC is observed only in humans. In human activity, the typical case of product manufacturing is tool-making for resource acquisition. This notable human behavior requires the combination of elemental objects or units made from elemental objects. In contrast, animals other than humans develop survival strategies without tool-making, in which object combination is not necessary.

#### Making a specific product

**Figure 7:**
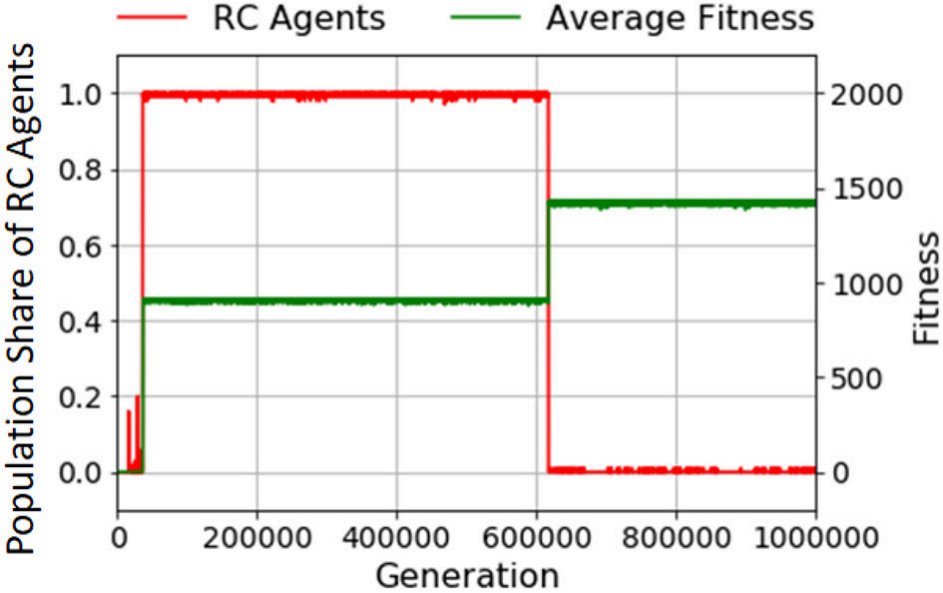
A typical example of transition of the population share of RC agents with *F*_II_. The x axis is generation. The y axis on the left is the population share of RC agents (red line), and that on the right is average fitness over the population (green line).

With the fitness function *F*_II_, Figure 6B demonstrates that the RC has appeared; it increased the average fitness when it appeared. It disappeared, however, with increasing the average fitness as shown in Figure 7 that depicts an example of the transition of the population share of RC agents in a typical run under *F*_II_. This phenomenon implies that RC makes it easier to discover a specific product than non-RC (a detailed explanation of this point is in the next paragraph). An agent using non-RC for a product obtains more fitness value than an agent using RC for the same product because RC requires longer manipulation steps than non-RC; and the opportunity for making products is limited by the upper limit of manipulation steps. Therefore, after the product is discovered, RC agents are taken over by non-RC agents. When the length of the gene (which is determined by *k* and *l*) is too long, it is hard for non-RC agents to take over from RC agents because the mutation is one locus per agent per generation. For example, converting an agent that performs a state transition shown in column 2 row 3 in Figure A1 in Appendix (RC) to one shown in column 1 row 1 (non-RC) needs to switch four loci.

The fitness landscape of *F*_II_ makes hill-climbing evolution virtually impossible and makes it hard to discover a specific product *x* for earning fitness. We employed the adaptability of RC by providing it with multiple routes to increase the discovery rate of a specific product. When the agent makes a specific product *ABABAB* (if only non-RC agents without stacks exist) the production of this specific product is unique because the elements must be obtained in exactly the same order from left to right of the specific product, as shown in the top left of Figure A1 in Appendix. Therefore, the discovery rate of making a specific product is very low. In contrast, if RCs are possible, at most 25 methods for making the product are available. Thus, the discovery rate greatly increases. Additionally, multiple methods to make a specific product promote robustness against failure in making processes (for which a detailed explanation is in section Effect of Failure Rate of Combination on Recursive Combination). In summation, the first adaptability of RC is diversifiability of production methods.

**Figure 8:**
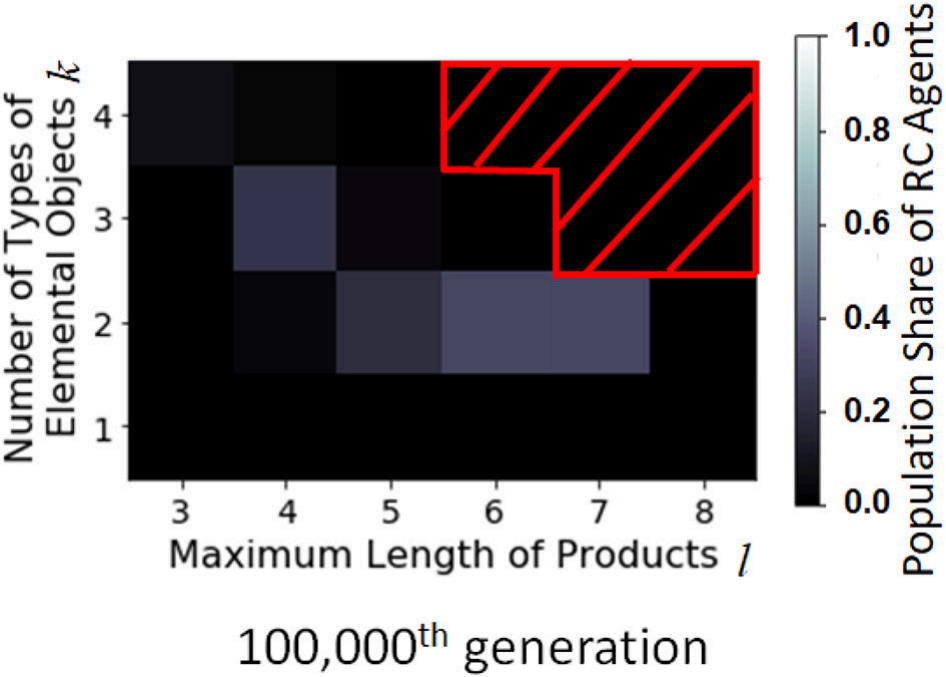
Distribution of the population share of RC agents with *F*_II_ in the combinatorial space parametrized by *l* and *k* at the 100,000th generation. The horizontal axis is the maximum length of product, *l*, the vertical axis is the number of types of elemental objects, *k*, and the brightness is the population share of RC agents (average of 200 runs). The part masked by the red oblique lines is the point where simulation results are not available due to limited computational power.

The number of production methods using RC depends on the size of the combinatorial space. Figure 8 shows the population share of RC agents in a combinatorial space parametrized by the number of types of elemental objects *k* (vertical axis) and the maximum length of products per product *l* (horizontal axis). When *k* = 2, the combinatorial space is larger than when *k* = 1, the RC agents evolve more frequently than when *k* = 1; however, if the combinatorial space is too large, the agents cannot discover the production process of a specific product until the 100,000th generation.

#### Making products as diversified as possible

**Figure 9:**
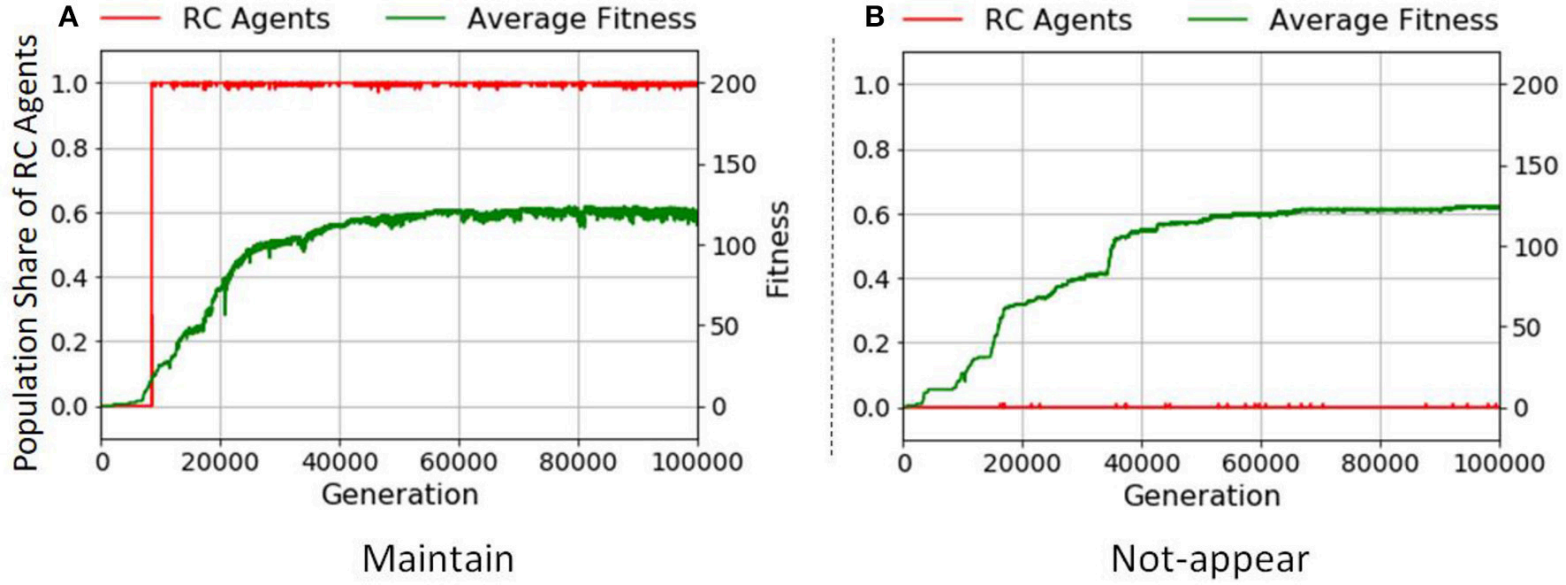
Examples of the transition of population share of RC agents in *F*_III_. **(A)** A case where the RC agents are maintained, and **(B)** a case where the RC agents do not appear. The x axis is generation. The y axis is the population share of RC agents.

In an environment fostering diversified products, RC evolves most in the three fitness functions as shown in Figure 6C compared with other cases (Figures 6A,B). Figure 9 shows typical examples of the transition of the population share of RC agents in two runs with *F*_III_. In this fitness function, the maximum fitness depends on the size of combinatorial space. If the upper limit of manipulation steps is sufficient for making all types of products, both RC and non-RC can earn the maximum fitness. Therefore, the RC agents or the non-RC agents can be maintained once either achieved the maximum fitness.

**Figure 10:**
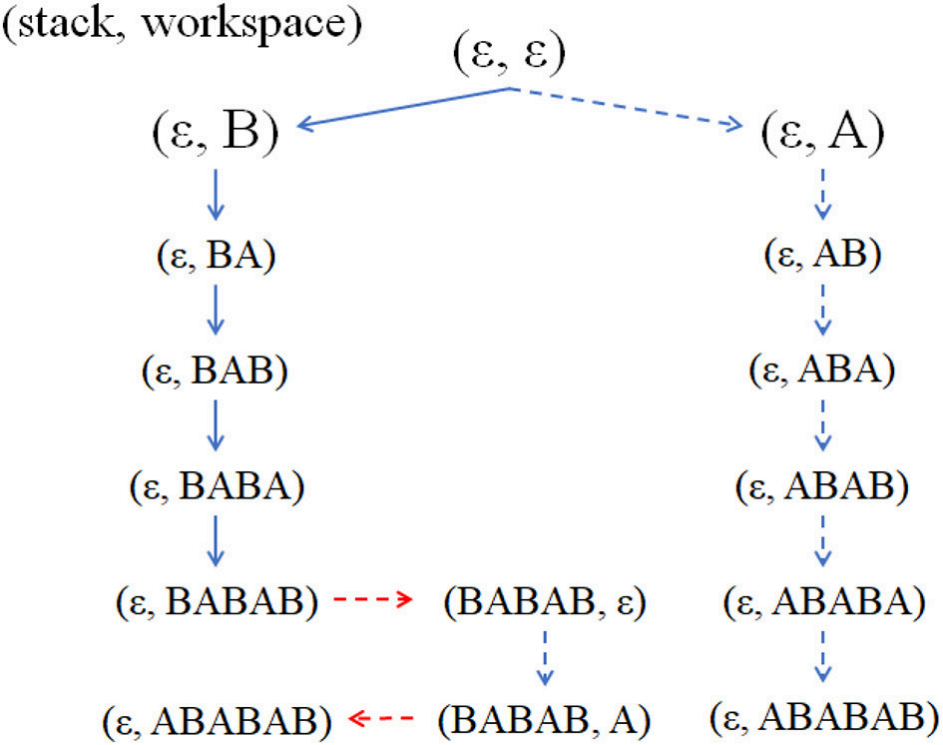
Examples of state transitions (portion) using Get action (blue arrows) and using stack actions (Push and Pop, red arrows). The notation (x, y) is that x is the stack state and y is the workspace state. The broken arrows are actions whose corresponding loci are not turned on. The vertical arrows represent Get actions, and the horizontal arrows Push (rightward) or Pop (leftward) actions.

RC agents more frequently appears than other fitness functions because the production method using RC to make new products can evolve by less loci change than that using only non-RC. We explain this difference using Figure 10. For example, when an agent can already make *BABAB* as shown by solid arrows in the left branch, the agent evolves to make *ABABAB* by three loci changes represented by the broken arrows which depict the RC production method. These changes are much fewer than evolving to make the product only with the non-RC making method as shown in the right branch (6 loci changes). Therefore, agents to make new products using RC method are more easily attainable than those using non-RC method in evolutionary process. Further those that make new products earn more fitness than their ancestral agents. Thus, RC agents can appear and spread more rapidly than non-RC agents with *F*_III_. The second adaptability of RC is diversifiability of product.

**Figure 11:**
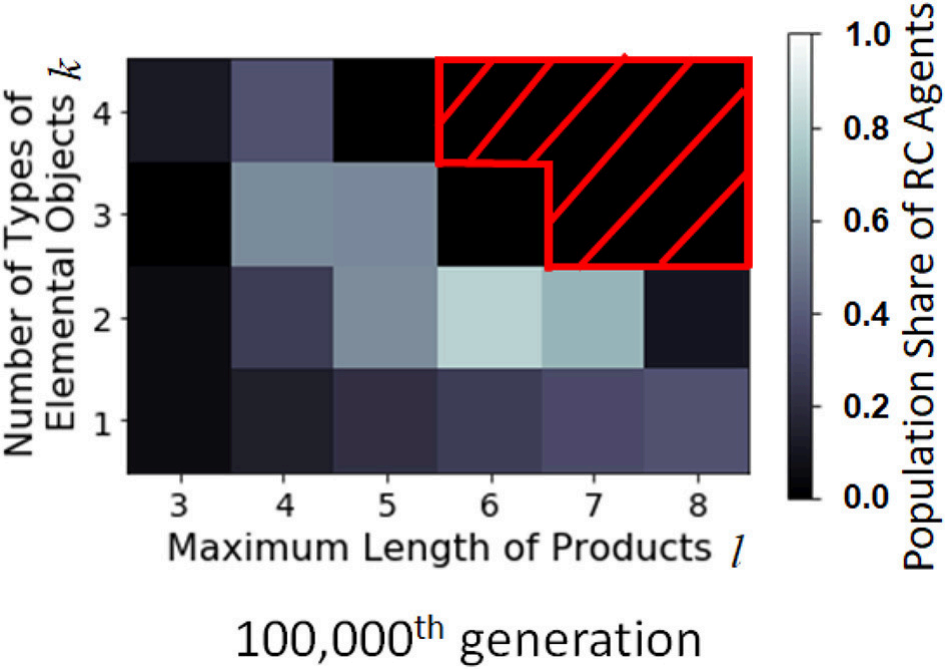
Distribution of the population share of RC agents with *F*_III_ in the combinatorial space parametrized by *l* and *k* at the 100,000th generation. The horizontal axis is the maximum length of products, and the vertical axis is the number of types of elemental objects, and the brightness is the population share of RC agents (average of 200 runs). The part masked by the red oblique lines is the point where simulation results are not available due to limited computational power.

The effect of the size of combinatorial space was investigated. Since the RC production method is more effective in searching production space than non-RC, the RC agents are more likely to evolve when the combinatorial space is large enough as shown in the center part of Figure 11. However, if the combinatorial space is very large, such as *k* = 3 and *l* = 6, the making processes of products are difficult to find, and the RC agents are not likely to appear by the 100,000th generation.

### Factors affecting the evolution of recursive combination

In the previous settings of the fitness functions, we identified two adaptabilities of RC: the diversifiability of production methods and the diversifiability of product. From these results, in this section, several factors that may affect the evolution of RC are introduced. The factors are the cost of manipulation and the failure of combination. RC exhibits a disadvantage when tool-making requires energy. In contrast, the diversification of production methods is useful for failure in object combination. As a result, these factors affect the evolution of RC. The evolutionary scenario of RC is expected from these effects.

#### Effect of manipulation cost on recursive combination

RC requires more manipulation steps than non-RC. We did not consider the cost incurred to perform operations in the simulation described in the previous section. If RC is costlier than non-RC, how does their evolution change? In order to find answers, we modified the fitness functions *F*_II_ and *F*_III_ as follows:

(5) $$\begin{array}{l} F_{\text{II}}^{{\;}^{\prime }}=\underset{x}{\sum }\begin{array}{c} \frac{n_{x}^{i}\left(t\right)}{m_{x}^{i}{\left(t\right)}^{c}} \end{array}, \end{array}$$

(6) $$\begin{array}{l} F_{\text{III}}^{{\;}^{\prime }}=\underset{x}{\sum }\begin{array}{c} \frac{\delta \left(n_{x}^{i}\left(t\right)\right)}{m_{x}^{i}{\left(t\right)}^{c}}, \end{array} \end{array}$$

where $m_{x}^{i}\left(t\right)$ is the manipulation steps required to make the product *x* at each production for (5) and at its first production for (6) and the parameter *c* regulates the effect of the cost.

**Figure 12:**
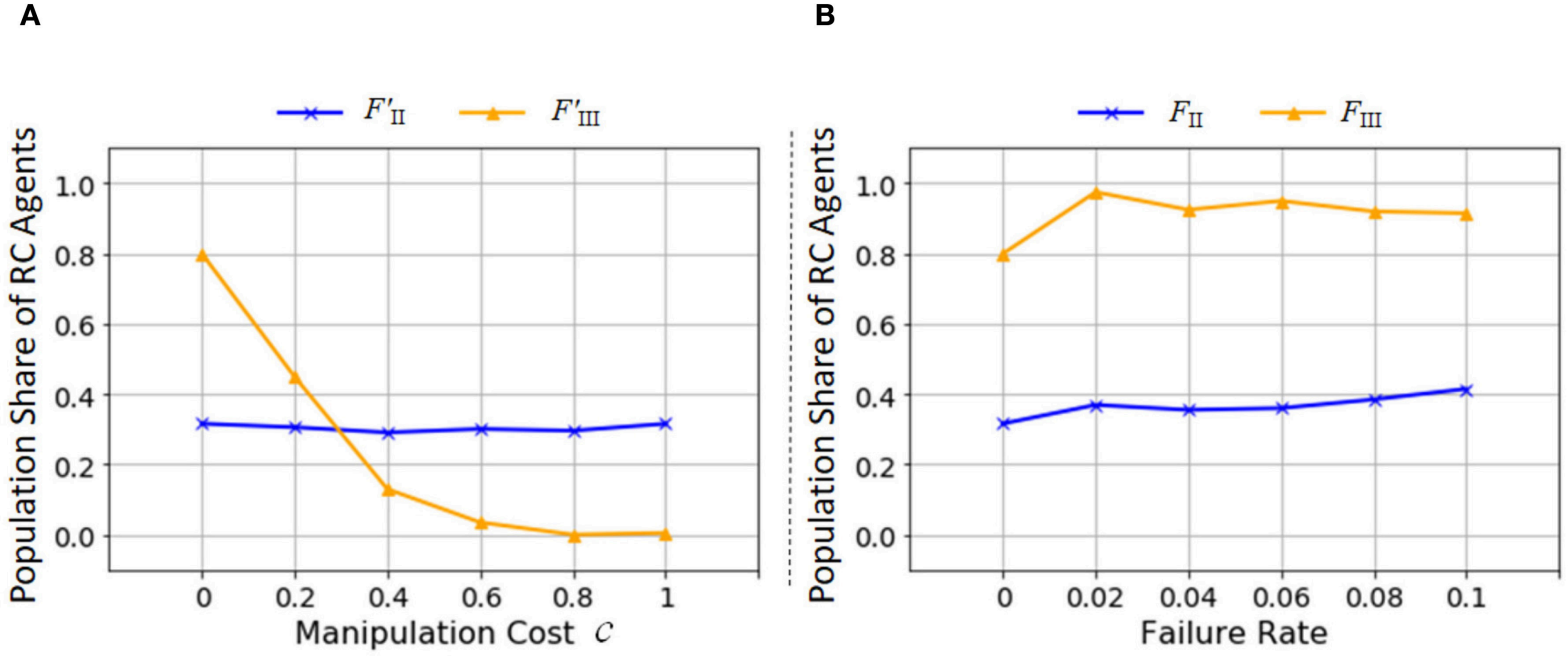
Parameter dependencies of the population share of RC agents at the 100,000th generation with *F*′_II_ or *F*_II_ (blue), and *F*′_III_ or *F*_III_ (yellow) (average of 200 trials). The x axis is **(A)** the parameter *c* for controlling the manipulation cost and **(B)** the failure rate. The y axes are the population share of RC agents.

The agents incur the manipulation cost when they perform Get, Push, and Pop actions. Figure 12A illustrates the effect of the manipulation cost on the population share of RC agents. It is naturally understandable that increasing the manipulation cost made the evolution of RC more difficult with *F*′_III_ since RC requires more manipulation steps than non-RC. Even if an agent makes many types of products, the fitness is discounted at the cost of production depending on manipulation steps. However, with *F*′_II_, the manipulation cost does not influence the evolution of RC. Since the fitness landscape of *F*′_II_, and *F*_II_ as well, is not a hill-climb type but discrete, the difference of fitness values of the fitted traits is hard to affect the possibility of takeover from RC to non-RC agents (A detailed explanation is provided in section Making a Specific Product, paragraph 1).

#### Effect of failure rate of combination on recursive combination

In the fitness function *F*_II_, we expected that the multiple production methods by RC would promote robustness against failures in production processes. We introduced the failure of combination action into the model to confirm this expectation. With a constant probability, the agents fail to combine objects using Get or Pop action, and the state of the workspace becomes empty. This modeling expresses that a product is broken due to a failure of combination. The fitness functions are the same as the Equations (3) and (4). Figure 12B shows that the probability of appearance of RC increase gradually with increase in the failure rate of the fitness function *F*_II_. In *F*_III_, the population share of RC agents rise when the failure rate is not zero but decrease slightly with a larger rate of failure. These increases are explained by the function of stack to keep a combined object. If an agent fails to make a product on the way of production, the agent does not have to return to the initial state but can restart from a production step when a partial product is kept in the stack. This function of stack realizes the diversification of production methods, but is not so strongly effectual for robustness. Actually, it is not so successful for higher failure rate in *F*_III_, the higher is the failure rate and the longer is the manipulation steps for a product, the more difficult to complete the production process of the product. Thus, the population share of RC agents decreases with larger failure rate in *F*_III_.

## Discussion

In this section, we mainly discuss the implication of each simulation result and its application to human evolution and language from the viewpoint of producing action sequences such as making tools. First, from the simulation results of *F*_II_ and *F*_III_, the adaptability and evolvability of recursive combination are considered. Next, a possible evolutionary scenario of recursive combination in human history is provided and supported with evidence from anthropology and archeology. Then, we speculate that recursive combination realizes flexibility of interpretation (that corresponds to diversifiability of production methods and of language products or expressions) and a driving force to diversify concepts and culture. Finally, we discuss the origin of recursive combination and recursive syntax by comparing two hypotheses (1) evolution of recursive combination via action control and (2) boot-strapping of recursive syntax via recursive intentionality.

### Adaptability of recursive combination

In an environment in which making a specific product with a complicated sequence is adaptive, production methods using recursive combination are discovered frequently (section Making a Specific Product). Additionally, the availability of multiple production methods for one tool is a workaround for inaccurate and/or irreversible manipulation. The greater access an agent has to multiple production methods the better that agent can make tools with increased stability (section Making a Specific Product); therefore, agents using recursive combination evolve faster than those that do not. When an agent must use many types of objects for product-making or must undergo a long process to make products (section Effect of Failure Rate of Combination on Recursive Combination), the frequency of failure derives from increase in inaccurate or irreversible manipulation; thus, diversifiability of production methods using recursive combination is effective.

In an environment in which making products as diversified as possible is adaptive, an agent searching for a production method that reuses existing methods can obtain relatively larger fitness than those who search for an all-new production method (section Making Products as Diversified as Possible). Therefore, the agent using recursive combination passes on its gene more easily than others. This adaptability is the diversifiability of products. In other words, recursive combination may have diversified the types of product in material culture beginning from stone tools. Human beings have diversified and complexified technology from the early stone age to the present. Arthur and Polak (2006) show that recursive combination of modularized technologies helped to identify more complex structures in a vast searching space. If the agents incur high manipulation costs, the adaptability of the diversifiability of products does not work (section Effect of Manipulation Cost on Recursive Combination).

Although we have already attempted other variants of this model, the approximate results of simulation (adaptability of recursive combination) did not change. The adaptability of recursive combination will not be altered by adopting a learning algorithm such as a neural network instead of GA, since learning algorithms do not influence the size of the learning space for the production procedure. This expectation, however, has to be checked in future research.

### The evolution of recursive combination in human history

How is a condition formed in which recursive combination is adaptive? From the results of the simulation with *F*′_II_ and *F*′_III_ (Figure 12A), when the manipulation cost is applied, recursive combination is used more easily and with a lower cost. As we introduced in section Results, recursive combination is not common in animal behavior; we assume that this strategy is costly and not adaptive in most environments. Consequently, we must identify the environmental conditions that promote the evolution of recursive combination while considering the existence of manipulation cost. Manual dexterity may be a key factor to performing significant object manipulations with decreased cost. Development of dexterity can lower manipulation cost at product-making.

Is there any archeological evidence in human evolutionary history corresponding to our proposal? In fact, the morphology of the early hominin's hand 3.00 mya acquired forceful opposition of the thumb, that is, an opposable thumb with the ability to exert forceful precision and power “squeeze” gripping (Skinner et al., 2015). Moreover, by 1.42 mya the hominin's hand had essentially evolved into the form of the modern human hand (Ward et al., 2013), in particular in terms of the distinctively human arrangement of the wrist associated with enhanced hand function when making and using tools. This evidence implies that early hominins might have been able to use their hands as dexterously as modern humans. According to other archeological evidence, tool use started around 3.39 mya (McPherron et al., 2010); tool-making around 2.60 mya (Plummer, 2004); and the recursive combination of objects around 0.28 mya (Moore, 2010). When the cost of object manipulation was high, recursive combinations could not have been maintained (section Effect of Manipulation Cost on Recursive Combination); this parallels the reasons that recursive combination is difficult to observe in animals, that is, its disadvantages (energy loss, manipulation injuries due to mistakes, etc.) are greater than its benefits.

Based on this account, we speculate on the possible evolutionary process of recursive combination. First, hominins came to use stone tools more frequently. This led to the evolution of hands and fingers to become dexterous enough to make superior tools that could survive repeated use. This dexterity helped decrease the cost of object manipulation and increase the chance of tool-making by reducing the steps to make each tool. When certain complicated tools were produced, recursive combination emerged as an adaptability to avoid failure in making these tools through diversification of production methods. Finally, these agents used their developing ability of recursive combination to develop various new tools, showing adaptability by diversifiability of products.

Diverse products can be made without recursion, and the recursive and non-recursive combinations can produce the same set of products. We argue, however, that recursive combination can increase the efficiency of product-making. If agents use non-recursive combination only, they make products through specific procedures. If they use recursive combination as well, they can create a variety of products from the combination of partial modules, and the creation procedure becomes flexible; thus, the success and discovery rates of production are improved. We showed that improving the success and discovery rates contributes to the successful diversification of products. Hominins could create a variety of products from the combination of partial modules or procedures in actual behavior of making stone tools (Moore, 2010, 2011; Stout, 2011).

### Recursive combination in language

Let us now consider whether the adaptability of recursive combination (shown by this simulation and explained by the speculative evolutionary account above) can also be demonstrated in language. Recursive combination in language, that is, a syntactic operation, is used to generate hierarchically structured symbol sequences. In our simulation, object manipulation and product manufacturing are modeled on the lines of an agent combining elemental objects represented by a letter such as *A* and *B*, or a combined object by concatenating letters, such as *AB* or *ABC*. If this model applies to language, elemental objects are lexical items, and products are sentences. For instance, when non-recursive combination is performed, words are combined repeatedly, e.g., the agent combines a word *book* and a word *club* to a word *child*. When recursive combination is performed, combined words (phrase) combine to form another word or phrase, e.g., the agent combines words *child* and *book* and then combines it with *club* to form *child book club*.

Diversifiability of production methods by recursive combination in language is presumed to encompass the making of multiple hierarchical structures, because various combination procedures can be of utility. This diversifiability assists plentiful interpretations to one expression. In linguistic communication, the interpretations of a sentence depend not only on sequential order but also on hierarchical structures that are not directly disclosed to receivers. The multiple hierarchical structures may cause ambiguity in meaning sharing when hierarchical structures represent meanings as the notable characteristic of human language, which is known as structural dependency.

Diversifiability of products by recursive combination in language then entails generating various expressions or ideas, because various possible combinations of lexical items can be assumed by this adaptability. In this way, recursive combination enables and requires the creation of new expressions and concepts by combining symbols.

Taking together the two types of diversifiability described above, we introduce a concept called *co-creation*. Making a hierarchical structure by combining symbols does not merely produce an internal expression but constructs a hierarchically structured concept that leads to the creation of a new, sometimes fictitious, concept that can attain a socially shared reality via linguistic communication. At the same time, however, the interpretation of these hierarchically structured sequences remains potentially ambiguous, enabling message receivers (as well as senders) to produce personal, sometimes creative, conceptual structures. In short, the interaction between senders and receivers promotes creativity in both parties. Our premise is that the adaptability of language is in co-creation. Co-creation is not necessarily a creative activity through actual collaboration. The viewpoint of co-creation, integrating two different functions (communication and thinking) can explain the reality and nature of humans and the human cultures they have cumulatively created (or, that have cumulatively evolved). Humans create and share new concepts via linguistic communication and produce higher-level concepts. *Money*, a symbolic concept socially created and shared, is a good example. We mutually believe that it mediates exchange among us, measures value, and makes it possible to store wealth—and so it does, based on this belief and on the new conceptual structures supported by this belief, such as banks, bonds, capital markets, and the global economy. In this way, novel concepts emerge and are *realized* through the interaction of the thinking function and the communication function. The cultural explosion and the spread of mankind all over the world around 50–100 Kya (Mithen, 1996) can be considered as having been brought about by co-creation through linguistic communication.

On the other hand, if new concepts and expressions continue to be created only in a certain group, cultural isolation may occur between that group and other groups. In particular, higher-level, abstract concepts that do not have concrete existence and are not grounded in any physical object, are often very difficult to interpret and share due to lack of appropriate underlying concepts and linguistic means to convey their meaning. This difficulty of mutual understanding is probably a major cause of cultural conflict.

### Origin of recursive combination and recursive syntax

In the introduction, we mentioned two reasonable hypotheses, origin of recursive combination via action control (Fujita, 2009, 2016) and boot-strapping of recursive syntax via recursive intentionality (Stiller and Dunbar, 2007; Oesch and Dunbar, 2017). In this subsection, the possibility of integrating these two hypotheses will be discussed as a future research. Recursive combination in object manipulation is to combine combined objects. Recursive intentionality has a structure that embeds a subject into a subject. It might be that these two hypotheses describe similar evolutionary scenarios of two different abilities.

In our simulation model, recursive combination needs a stack to store an object temporarily. In human cognition, this function for temporal storing is implemented by working memory (Baddeley, 2000, 2007). Working memory is an important faculty for higher order general cognition and behavior in humans, i.e., complicated action planning, presence of intentionality, and generation or recognition of other physical or conceptual structures. Therefore, we should consider an evolutionary process of working memory in human history.

Stout (2011) analyzed the production methods of stone tools that required complicated action planning, both the Oldowan and Acheulian types, and illustrated the methods using a tree diagram (Stout, 2011, Figure 1). The analyses of stone tool-making in Moore (2010, 2011) and Stout (2011) are almost the same. The notable point of Stout's (2011) analysis is using a tree diagram with dominance relationship in hierarchical structures. According to this analysis, the process of production of Oldowan tools required several steps of action: procurement of materials (for stone core and hammer stone) of appropriate size, shape, and composition; examination of the core; selection of target point to strike; positioning and fixing of the core; selection of hammerstone grip; and finally, accurate striking. These manipulations can be expressed by a tree diagram that has sixth order nesting. Unlike Moore (2010, 2011), Stout (2011) argued that the production method of Oldowan tools has discrete infinity that leads to the hierarchical structure of language. In the production method of the Acheulian type, Stout pointed out that the action sequence for achieving sub-goals was incorporated recursively into a higher order goal since the process of making a stone flake was included in the higher order intention of making stone flakes.

Arbib (2011) simplified the analysis of Stout's (2011) tree diagram from the viewpoint of working memory and re-interpreted the sixth order tree diagram in the production of Oldowan tools to five working processes. The five processes correspond to the following questions that stone tool-makers must answer: (1) Do I have a hammerstone? (2) Do I have a core? (3) Is there an available affordance for flake detachment? (4) If so, proceed with flake detachment. (5) If not, back up as far as needed. For Acheulian tools, Arbib insisted that automatization of the action sequence (working memory becomes needless) was essential because a complicated action sequence for stone flaking was incorporated into a subordinate component of the production of a stone tool. These studies argued that maintaining and combining sub-goals or sub-ordinate processes were essential for goal-directed action sequences that was a remarkable feature of Acheulian stone tools. Therefore, it is highly possible that the ability of recursive combination appeared in the age of Acheulian at the latest. In our simulation, a learning process such as Arbib's “automatization” is not implemented. We will clarify the relation of recursive combination and automatization as a future work by employing simulations with learning algorithms.

Mentalizing also needs working memory to maintain the mental state of others who have intentionality, such as *Simon believes that Martin thinks that Charlotte supposes that Jane knows that Simon thinks …*. Some studies show that mentalizing is limited to around the fourth or fifth order by working memory requirements (Stiller and Dunbar, 2007; Oesch and Dunbar, 2017). Oesch and Dunbar (2017) experimentally suggest that from first to fifth-order intentionality is necessary to assist the processing of simpler syntactic structures, but beyond fifth-order intentionality the cognitive scaffolding is provided by recursive syntax. We may apply this suggestion to the hypothesis of the origin of recursive combination via action control. Namely, lower-order recursive combination is necessary to assist the processing of simpler syntactic structures, but for more complicated action planning the cognitive scaffolding is provided by recursive syntax. It is assumed that two cognitive abilities, recursive combination and inference of intentionality, evolved separately then they were integrated to create diverse and complicated hierarchical structures.

We do not claim to know the origin of recursive syntax. However, we argue that, if diversity, novelty, and robustness of production are required to survive or reproduce, recursive combination has adaptability in the various domains, and the ability of recursive combination needs working memory. It does not matter whether it originates from action control, social cognition, or others.

## Conclusion

Adopting the hypothesis that recursive combination of object manipulation is the precursor of the syntactic ability intrinsic to human language, we developed an evolutionary simulation of product-making to clarify the adaptability of recursive combination in human evolution. In our study, a recursive combination, which is considered as a unique human ability, was modeled as a recursive combination in action grammar.

The main finding reported by this study, as evidenced by an evolutionary simulation, *is that the adaptability of recursive combination increased the rate of discovery and success at product making by diversifying production methods* and therein *increased fitness by diversifying products*. We argue that recursive combination may have evolved to become a consistent feature of human nature, through the production and use of tools that was later generalized to many aspects of human cognition, including human language. Effectually, this may be part of the explanation as to how and why recursive combination evolved to become a consistent feature of human language, and not of other animal communication systems.

## Author contributions

GT designed the study, analysis of data, and wrote the initial draft of the manuscript. TH contributed to designing of the study, interpretation of data, critically reviewed and assisted in the preparation of the manuscript. All authors approved the final version of the manuscript, and agree to be accountable for all aspects of the work in ensuring that questions related to the accuracy or integrity of any part of the work are appropriately investigated and resolved.

### Conflict of interest statement

The authors declare that the research was conducted in the absence of any commercial or financial relationships that could be construed as a potential conflict of interest.

## Figures and Tables

**Figure A1A:**
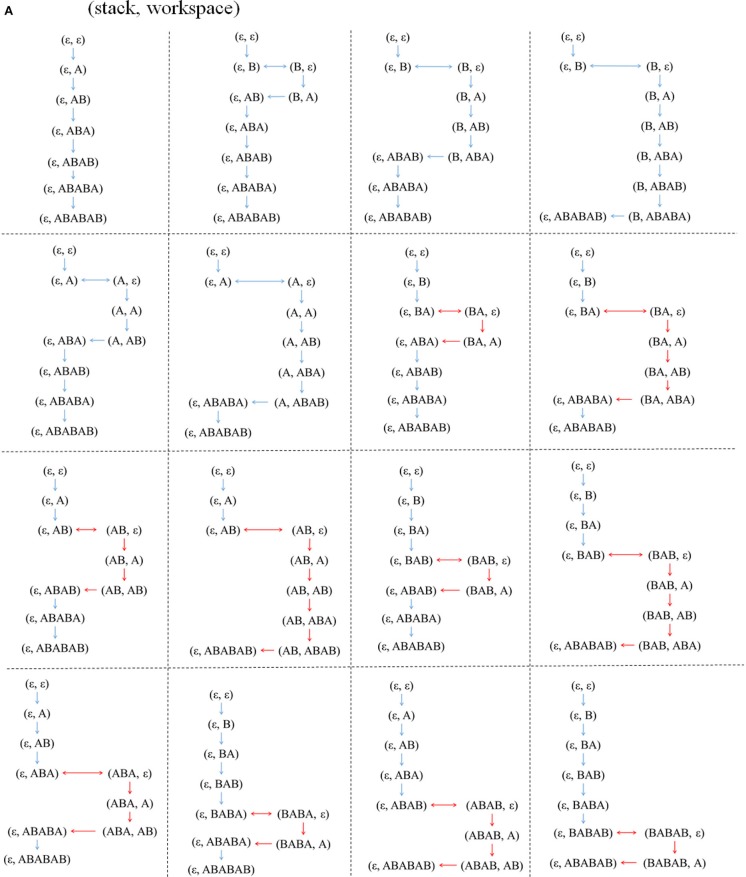
Examples (portion) of state transitions to make a product *ABABAB* using non-RCs (blue arrows) and RCs (red arrows). The notation (x, y) means that x is the stack state and y is the workspace state. Vertical arrows represent Get actions, and horizontal arrows represent Push (Rightward) or Pop (Leftward) actions. The table has no particular order. In case of other product than *ABABAB*, the ratio of state transition using RC changes. State transitions using the stack more than once are omitted.

**Figure A1B:**
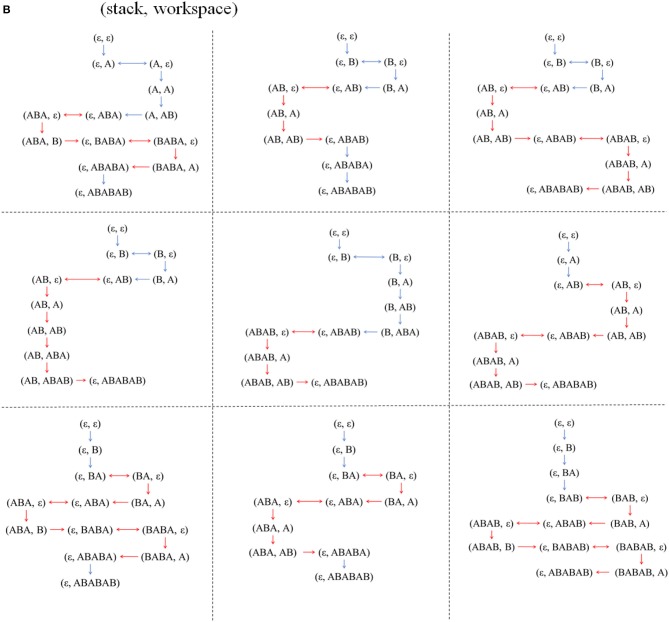
Examples (portion) of state transitions using the stack more than once to make a product *ABABAB* using non-RCs (blue arrows) and RCs (red arrows). The notation (x, y) means that x is the stack state and y is the workspace state. Vertical arrows represent Get actions, and horizontal arrows represent Push or Pop actions. The table has no particular order. In case of other product than *ABABAB*, the ratio of state transition using RC changes.

## Footnotes

^1^The regulatory locus was introduced to reduce computational time. We confirmed that there is no change in simulation results using a model without the regulatory locus for stacks.

## Appendix

Figure A1 is a summary of state transitions of workspace and stack to make a specific product *ABABAB*.
